# Epidemiology of inflammatory bowel disease-associated anemia in children and adolescents from 1990 to 2021: a global, regional analysis from the Global Burden of Disease Study

**DOI:** 10.3389/fped.2025.1651612

**Published:** 2025-10-30

**Authors:** Chenjing Xu, Xiaqiong Mao, Ying Xu, Xiufang Cui

**Affiliations:** Department of Gastroenterology, Nanjing First Hospital, Nanjing Medical University, Nanjing, Jiangsu, China

**Keywords:** inflammatory bowel disease, anemia, global burden of disease, children, adolescents

## Abstract

**Objective:**

Inflammatory bowel disease (IBD)-associated anemia cannot be ignored, as it can affect the growth and development of children and adolescents. This study aimed to report the global, regional, and national disease burden of IBD-associated anemia in children and adolescents.

**Methods:**

Data on the prevalence of IBD-associated anemia and years lived with disability were obtained from the Global Burden of Disease Study 2021. Joinpoint regression analysis, age–period –ohort modeling analysis, and decomposition analysis were performed. Finally, an ARIMA model was established to predict the disease burden up to 2041.

**Results:**

Anemia associated with IBD was most prevalent among adolescents aged 15–19 years, with significantly higher prevalence in females [11,487.26, 95% uncertainty interval (UI) = 8,826.54, 14,622.31] than in males (6,677.01, 95% UI = 5,179.56, 8,498.89). The age-standardized prevalence rate (ASPR) was highest in moderate anemia (17.5561, 95% UI = 14.9597, 20.8729). Joinpoint regression analysis suggested the ASPR of IBD-associated anemia in both males and females increased from 2005 to 2010 and then decreased after 2010. In the decomposition analysis, aging and population growth were the main drivers of changes in the prevalence of IBD-associated anemia. ASPR exhibits a fluctuating trend in males from 1990 to 2041 and is projected to remain stable in females from 2027 to 2041.

**Conclusion:**

The ASPR of IBD-associated anemia in children and adolescents increased from 2005 to 2010 and then decreased after reaching a peak in 2010. In the future, IBD-associated anemia will continue to pose a significant public health problem.

## 1 Introduction

Inflammatory bowel disease (IBD) is one of the most common chronic inflammatory diseases of the gastrointestinal tract and can be divided into two subtypes: ulcerative colitis (UC) and Crohn's disease (CD) (1). IBD is an immune-mediated disease with a lifelong tendency to relapse (2), with the age of onset being younger (3, 4). Approximately 10%–20% of patients with newly diagnosed IBD are under 20 years old (1). While the incidence of pediatric IBD is now stable in high-income countries, the incidence of pediatric IBD in newly industrialized countries such as South America, Eastern Europe, Asia, and Africa has increased rapidly in the past two decades (3, 5).

Clinical presentations of IBD in children and adolescents are variable. Growth failure, anemia, perianal disease, or other extraintestinal manifestations may be the sole prominent initial feature in 22% of pediatric patients (1, 6). Anemia is a prevalent complication of pediatric patients with IBD, affecting up to 75% of the patients (7, 8). The causes of anemia in patients with IBD include iron deficiency, vitamin B12 deficiency, and anemia of chronic disease (9), with iron deficiency being the most frequent. The leading causes of IBD-associated iron deficiency anemia include insufficient exogenous iron intake resulting from dietary changes, iron malabsorption following small intestinal lesions, and chronic blood loss due to intestinal mucosal ulcer injury (10). Similar to other chronic inflammatory diseases or infections, inflammatory responses can also lead to IBD-associated anemia (11, 12). Elevated levels of the inflammatory mediator IL-6 in patients with IBD cause elevated hepcidin levels, leading to retention of iron in macrophages and enterocytes, resulting in hyposalemia and iron-restricted erythropoiesis (13). Anemia obviously affects the quality of life in children and adolescents with IBD (14, 15). Other adverse consequences of anemia in children with IBD include more extensive disease, poor growth, decreased exercise tolerance, dizziness, fatigue, headache, shortness of breath, and restless legs syndrome, as well as worsening cognitive outcomes (14, 16, 17). The current epidemiological impact of IBD-associated anemia has been dramatically underestimated and has not been fully recognized and treated in children (8, 16). Without proper monitoring and treatment, IBD-associated anemia will not only cause a high negative impact on health but also bring a substantial economic burden to the modern health care system.

However, there is limited data on changes in epidemiological trends in rapidly industrializing emerging countries, especially in pediatric IBD-associated anemia (4). Identifying the different prevalences and prognoses of IBD in children and adolescents in different geographical regions can improve clinicians' awareness of the disease, intervene early, and reduce its impact.

Based on the Global Burden of Disease Study 2021 (GBD 2021) database, we report the global, regional, and national disease burden of IBD-associated anemia among children and adolescents from 1990 to 2021, including prevalence, epidemiology, and changes in years lived with disability (YLDs). In addition, the disease burden of IBD-associated anemia in children and adolescents to 2041 was predicted.

## 2 Materials and methods

### 2.1 Data sources

We obtained the data on children aged under 20 years across 204 countries and regions, together with global causes of IBD-associated anemia from 1990 to 2021 through further analysis. All data were obtained from the GBD study database (https://vizhub.healthdata.org/gbd-results/).

The GBD study quantified the burden of disease using a standardized approach and applied DisMod-MR as a non-fatal estimate for the standardization of primary tools. DisMod-MR is a Bayesian meta-regression tool used to evaluate prevalence incidence data for each non-fatal condition of a disease while enhancing agreement between epidemiological parameters (18).

### 2.2 Statistical descriptive indicators

Since GBD 2021 only provides data on the prevalence and YLDs, we used two indicators to estimate the level of prevalence: the crude prevalence rate and the age-standardized prevalence rate (ASPR). The disease burden was estimated by YLDs and the age-standardized YLD rate (ASYR) (18). All rates were age-standardized against the GBD world population and represented as per 100,000 population. We report the 95% UIs for all estimates (18).

### 2.3 Children and adolescents’ definition

In this study, children and adolescents (<20 years) were categorized into four groups based on the GBD study database and World Health Organization (WHO) definitions: <5 years, young children; 5–9 years, older children; 10–14 years, younger adolescents; 15–19 years, older adolescents (19).

### 2.4 Sociodemographic index

The sociodemographic index (SDI) is a composite measure of development used to assess a country or geographical area. It ranges from 0 to 1, with higher values indicating higher levels of comprehensive development. The SDI is scored according to three aspects: (1) lagged income distribution per capita; (2) average years of education over 15 years old; (3) total fertility rate (TFR) for females under 25 years of age. When per capita income is low, the average number of years of education is shorter, and countries with high TFR will be assigned a low SDI value. On the contrary, relative to other countries, per capita income is higher, the mean education period is longer, and the TFR will be higher in countries with lower SDI (4, 20).

The GBD database classified 204 countries and regions into 22 geographic regions and five categories of SDI levels: high SDI, high-middle SDI, middle SDI, low-middle SDI, and low SDI (21).

### 2.5 Data processing and analysis

#### 2.5.1 Joinpoint regression model

The Joinpoint regression model applies linear statistical models to evaluate the trends in the disease burden of IBD-associated anemia over time (22). We assessed the average annual percent change (AAPC) and 95% CI using Joinpoint regression analysis to determine the extent of temporal trends in prevalence. AAPC was calculated as a geometrically weighted average of different annual percent change values obtained from regression assessments (22). The software was provided by the Surveillance Research Program of the US National Cancer Institute (Joinpoint Trend Analysis Software Version 5.2.0).

#### 2.5.2 Age–period–cohort modeling analysis

The age–period–cohort (APC) model is a statistical tool used to analyze the epidemiology, demography, and sociology to parse and address the influence of three different time-related factors on specific outcomes or trends: age, period, and cohort effect. In this study, the APC model was used to analyze the global epidemic trends by age, period, and birth cohort from 1990 to 2021, and the specific methods were described in previous studies (23, 24). The results of the APC model include local losses in prevalence and local losses in age-standardized prevalence, which reflect average trends and the effect of birth rates across different years. Furthermore, the question includes a specific age ratio that corresponds to the size of the reference list to consider changes in time and the effect of age. Period and cohort expected effects indicate relative risk for each period and for men. The choice of reference period and stress level was random and not affected by the interpretation of the results (24).

#### 2.5.3 Decomposition analysis

Decomposition analysis is used to help understand how different factors contribute to the overall change. It can decompose the change in the number of people affected by disease over a period into changes in age structure, population growth, and epidemic trends. Decomposition of the prevalence of IBD-associated anemia according to age structure, population growth, and epidemiological changes can quantify their contribution to the overall effect (25).

#### 2.5.4 Autoregressive integrated moving average model

The autoregressive integrated moving average (ARIMA) model is used to predict the value of the future by time series analysis (26). The ARIMA model was established using the global prevalence of pediatric IBD-associated anemia from 1990 to 2021. It consists of three main steps: (1) data preprocessing of associated anemia, inspection when asked whether the sequence is stable, processing non-stationary sequence, and white noise testing; (2) model identification, model parameter estimation, combined with the red pool information criterion and Bayesian information criterion type and select the optimal model parameters; (3) inspection and prediction, the use of Ljung–Box inspection of residual white noise analysis for the optimal model of diagnostic test and finally forecast of future value.

All analyses were performed using R software (R version 4.4.0, Vienna, Austria). A two-sided *P*-value of <0.05 was considered statistically significant.

## 3 Results

### 3.1 Epidemiology of IBD-associated anemia in children and adolescents

**Figure 1:**
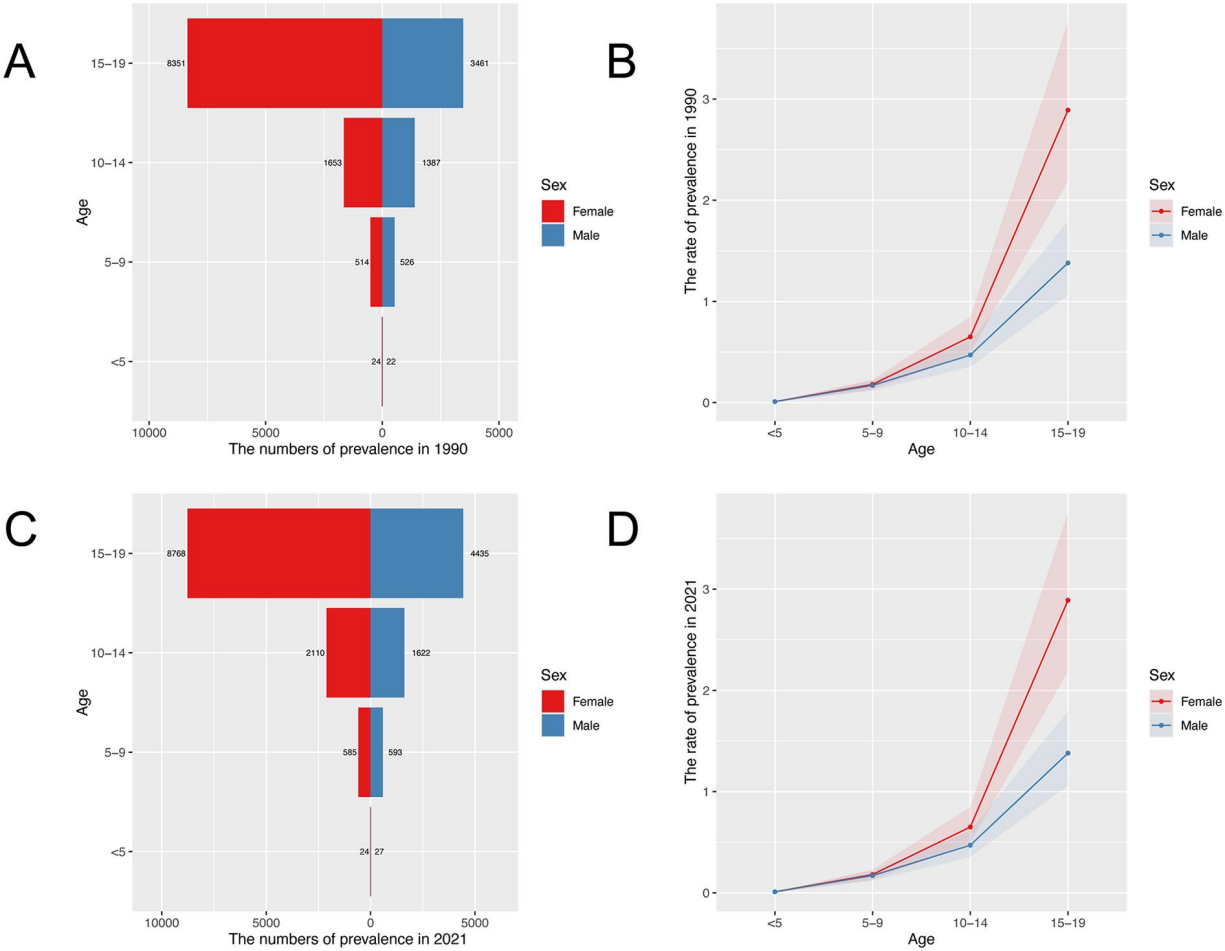
Age-specific prevalence numbers and prevalence rate for children and adolescents with IBD-associated anemia globally. **(A)** Global prevalence of IBD-associated anemia in 1990. **(B)** Global prevalence rate of IBD-associated anemia in 1990. **(C)** Global prevalence of IBD-associated anemia in 2021. **(D)** Global prevalence rate of IBD-associated anemia in 2021.

Figure 1 shows the prevalence numbers (Figures 1A,C) and rates (Figures 1B,D) of IBD for the different age groups in 1990 and 2021, respectively. IBD-associated anemia is more prevalent in children older than 10 years, with a rapid increase observed at aof 15–19. The prevalence numbers of females are obviously more than males at ages 15–19. The numbers of YLDs show similar trends by sex and age group (Supplementary Figure S1).

**Figure 2:**
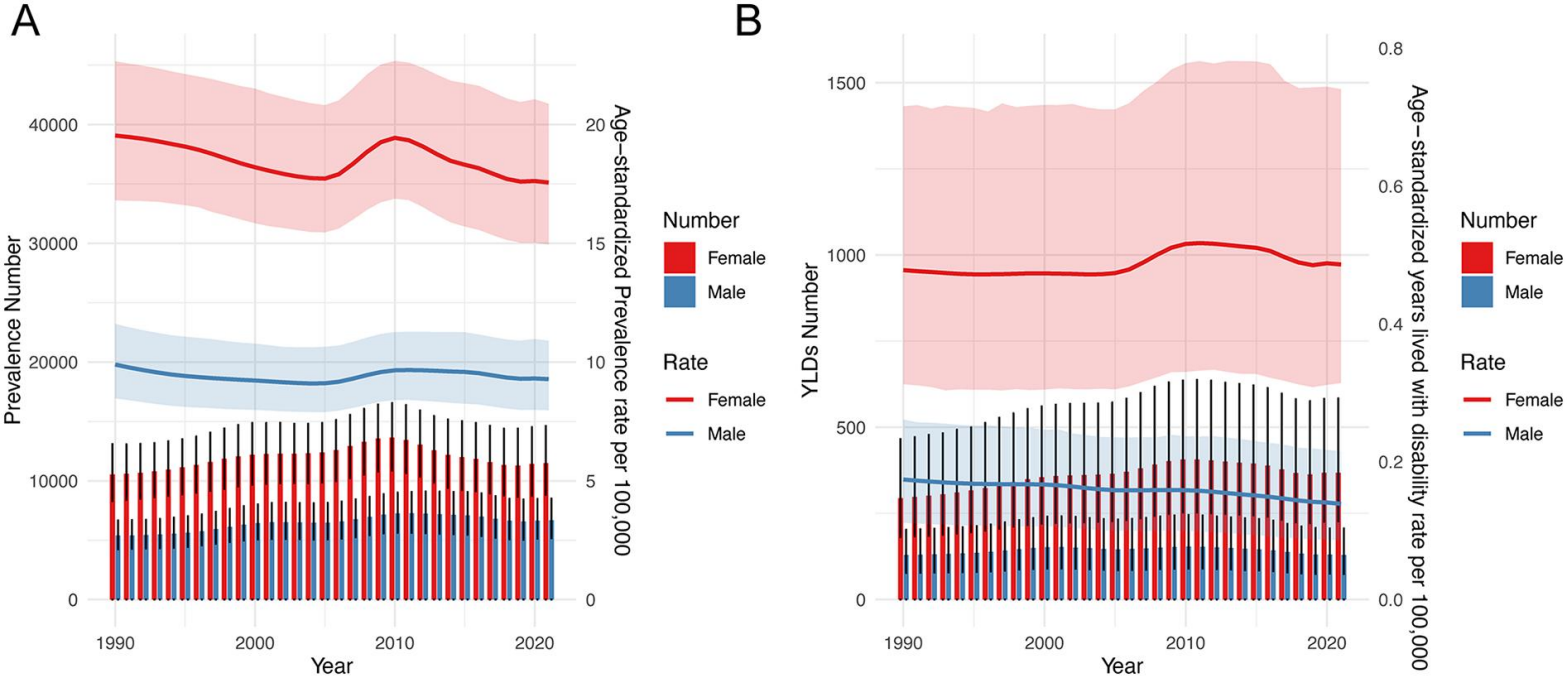
Trends in the prevalence number, YLDs, ASPR, and ASYR for children and adolescents of IBD-associated anemia by sex from 1990 to 2021. **(A)** Prevalence number and ASPR. **(B)** YLDs number and ASYR.

Figure 2 shows the trends in the prevalence and YLDs number, ASPR, and ASYR for children and adolescents of IBD-associated anemia by sex from 1990 to 2021. Prevalence increased briefly from 2005 to 2010 and then slowly declined. ASPR decreased from 1990 to 2005, then increased from 2005 to 2010, and decreased again from 2010 to 2021 (Figure 2A). ASYR consistently decreased in males but changed little in females over the 31-year period (Figure 2B). Notably, the prevalence number, YLDs number, ASPR, and ASYR of IBD-associated anemia were higher in females than those in males in all years.

The global prevalence number of IBD-associated anemia in children and adolescents was 18,164.27 [95% uncertainty interval (UI) = 14,057.30, 22,998.85] in 2021, and females (11,487.26, 95% UI = 8,826.54, 14,622.31) had a higher disease burden than males (6,677.01, 95% UI = 5,179.56, 8,498.89). The prevalence rate per 100,000 population with IBD-associated anemia in females also had a higher disease burden than males in 2021. Furthermore, IBD-associated anemia was mainly mild-to-moderate anemia. The ASPR per 100,000 population of IBD-associated anemia in children and adolescents was 13.3914 (95% UI = 11.5099, 15.8218) in 2021. Among anemia categories, moderate anemia showed the highest ASPR (17.5561, 95% UI = 14.9597, 20.8729) (details are shown in Table 1).

**Table 1:** Number, rate, and age-standardized rates of prevalence and YLDs in 2021 for children and adolescents with IBD-associated anemia globally.

		<20 years number (UI^a^)				<20 years rate per 100,000 people (UI)				Age-standardized rates per 100,000 people			
Sex	Measure	Mild anemia	Moderate anemia	Severe anemia	Total	Mild anemia	Moderate anemia	Severe anemia	Total	Mild anemia	Moderate anemia	Severe anemia	Total
Male	Prevalence	4,715.03 (3,633.37, 6,104.19)	1,850.14 (1,419.14, 2,322.81)	111.84 (82.19, 150.91)	6,677.01 (5,179.56, 8,498.89)	0.3471 (0.2675, 0.4493)	0.1362 (0.1045, 0.1710)	0.0082 (0.0061, 0.0111)	0.4915 (0.3813, 0.6256)	0.0317 (0.0111, 0.0714)	1.6199 (1.3788, 1.9109)	0.1938 (0.1621, 0.2302)	9.2794 (7.9744, 10.8999)
Female	Prevalence	5,464.24 (4,070.01, 7,210.03)	5,634.46 (4,408.91, 7,115.87)	388.55 (288.73, 495.58)	11,487.26 (8,826.54, 14,622.31)	0.4278 (0.3186, 0.5645)	0.4411 (0.3452, 0.5571)	0.0304 (0.0226, 0.0388)	0.8993 (0.6910, 1.1447)	0.0362 (0.0127, 0.0801)	7.1416 (6.0607, 8.4707)	0.5846 (0.4975, 0.6973)	17.5561 (14.9597, 20.8729)
Total	Prevalence	10,179.27 (7,877.87, 13,036.87)	7,484.60 (5,789.45, 9,390.67)	500.40 (377.00, 633.97)	18,164.27 (14,057.30, 22,998.85)	0.3862 (0.2989, 0.4946)	0.2840 (0.2196, 0.3563)	0.0190 (0.0143, 0.0241)	0.6891 (0.5333, 0.8725)	0.0317 (0.0111, 0.0714)	4.3970 (3.7563, 5.1866)	0.3896 (0.3341, 0.4607)	13.3914 (11.5099, 15.8218)
Male	YLDs	17.47 (6.10, 41.36)	95.32 (55.66, 144.65)	16.62 (10.51, 25.58)	129.41 (74.30, 206.04)	0.0013 (0.0004, 0.0030)	0.0070 (0.0041, 0.0106)	0.0012 (0.0008, 0.0019)	0.0095 (0.0055, 0.0152)	7.4658 (6.3610, 8.7766)	0.0827 (0.0522, 0.1263)	0.0288 (0.0188, 0.0428)	0.1389 (0.0859, 0.2153)
Female	YLDs	20.26 (7.03, 46.46)	290.29 (177.54, 465.83)	57.71 (36.22, 86.71)	368.26 (227.46, 583.87)	0.0016 (0.0006, 0.0036)	0.0227 (0.0139, 0.0365)	0.0045 (0.0028, 0.0068)	0.0288 (0.0178, 0.0457)	9.8299 (8.3706, 11.6897)	0.3635 (0.2294, 0.5621)	0.0866 (0.0554, 0.1256)	0.4863 (0.3144, 0.7405)
Total	YLDs	37.72 (13.10, 85.56)	385.61 (235.38, 614.03)	74.33 (46.72, 108.75)	497.66 (302.95, 784.98)	0.0014 (0.0005, 0.0032)	0.0146 (0.0089, 0.0233)	0.0028 (0.0018, 0.0041)	0.0189 (0.0115, 0.0298)	8.6048 (7.4222, 10.2418)	0.2239 (0.1416, 0.3427)	0.0577 (0.0374, 0.0835)	0.3133 (0.1994, 0.4744)

YLDs, years lived with disability; IBD, inflammatory bowel disease.

^a^ UI is the uncertainty interval, which reflects the certainty of an estimate based on data availability, study size, and consistency across data sources.

Table 1 also shows the YLDs numbers and ASYRs of IBD-associated anemia for children and adolescents in 2021. YLDs numbers of moderate anemia caused by IBD were both highest in females (290.29, 95% UI = 177.54, 465.83) and males (95.32, 95% UI = 55.66, 144.65). However, ASYRs showed diverse trends by sex, suggesting higher YLD rates of mild anemia.

### 3.2 Epidemiology of IBD-associated anemia by sociodemographic development in 2021

**Figure 3:**
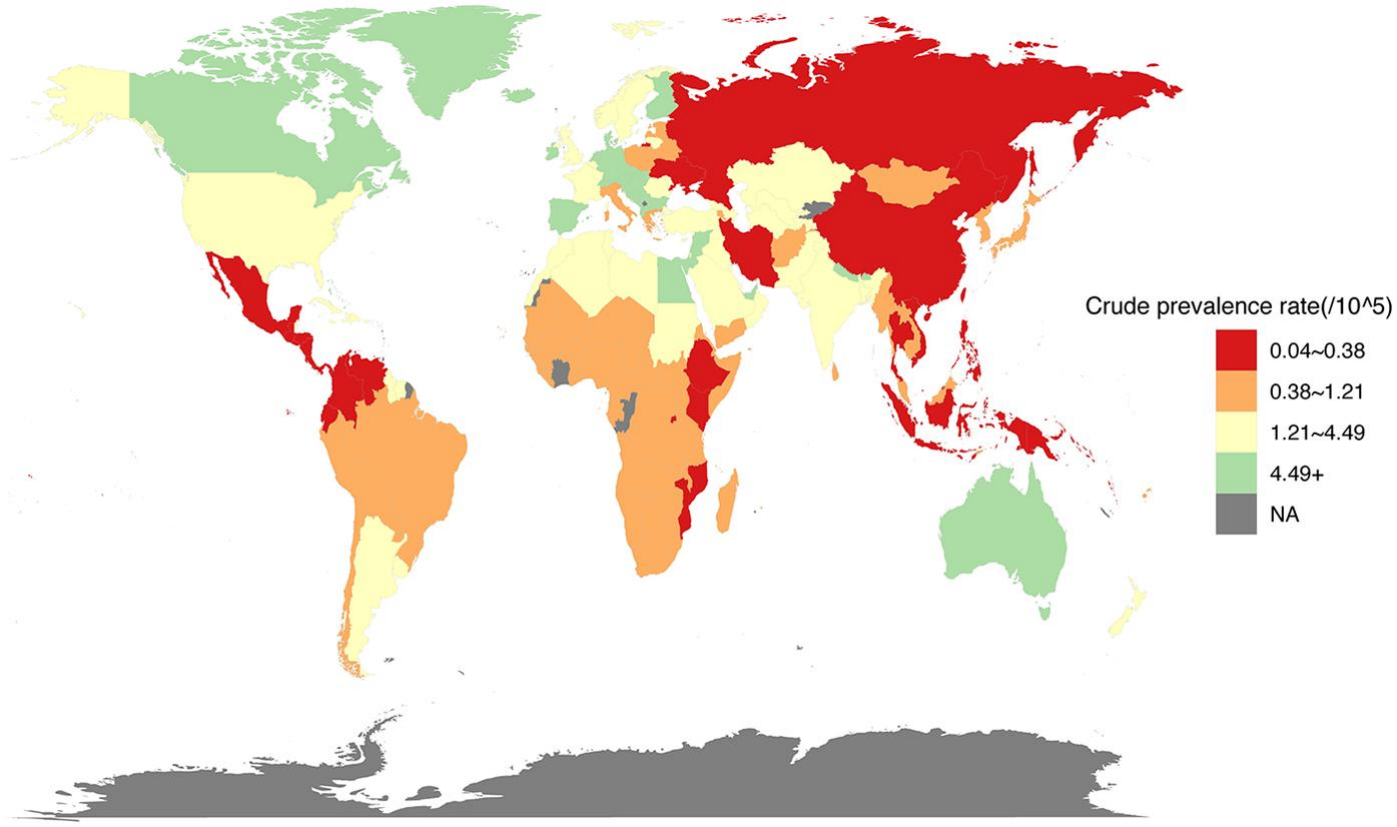
Prevalence rate (per 100,000 population) for children and adolescents of IBD-associated anemia, both males and females, for 204 countries and territories, 2021.

The global crude prevalence rate (per 100,000 population) for children and adolescents (both males and females) of IBD-associated anemia in 2021 is shown in Figure 3. The prevalence of IBD-associated anemia in children and adolescents in 204 countries and regions in the world in 2021 was obtained from the GBD database, and the corresponding prevalence was reflected in the form of heat maps.

The prevalence rate for children and adolescents (<20 years) of IBD-associated anemia per 100,000 population was highest for high-SDI countries (1.2326, 95% UI = 0.8601, 1.7068); countries in the low-middle (0.8397, 95% UI = 0.6496, 1.0800) and low (0.6179, 95% UI = 0.4707, 0.7966) SDI quintile exhibited a lower rate compared with high-SDI countries (Table 2). To gain a better understanding of the severity of anemia in children and adolescents with IBD globally and by SDI quintile, an anemia subgroup analysis profile of IBD-associated anemia was constructed. We observed that the highest prevalence rate and ASPR per 100,000 people were in the mild anemia group, the second was the moderate anemia group, and the severe anemia group was the lowest in different SDI countries, except low-middle SDI countries (details were shown in Table 2).

**Table 2:** Number, rate, and age-standardized rate in 2021 for children and adolescents of IBD-associated anemia by SDI quintile.

	<20 years number (UI^a^)				<20 years rate per 100,000 people (UI)				Age-standardized rates per 100,000 people			
Location	Mild anemia	Moderate anemia	Severe anemia	Total	Mild anemia	Moderate anemia	Severe anemia	Total	Mild anemia	Moderate anemia	Severe anemia	Total
Global	10,179.27 (7,877.87, 13,036.87)	7,484.6 (5,789.45, 9,390.67)	500.4 (377,633.97)	18,164.27 (14,057.3, 22,998.85)	0.3862 (0.2989,0.4946)	0.284 (0.2196,0.3563)	0.019 (0.0143,0.0241)	0.6891 (0.5333,0.8725)	8.6048 (7.4222,10.2418)	4.397 (3.7563,5.1866)	0.3896 (0.3341,0.4607)	13.3914 (11.5099,15.8218)
High SDI^b^	2,328.79 (1,573.24, 3,389.04)	526.72 (393.84,705.51)	13.01 (8.66,19.11)	2,868.52 (2,001.68,3,972.15)	1.0007 (0.676,1.4563)	0.2263 (0.1692,0.3032)	0.019 (0.0143,0.0241)	1.2326 (0.8601,1.7068)	24.3553 (20.6396,28.6248)	4.8606 (4.1187,5.7788)	0.1714 (0.1398,0.2121)	29.3873 (24.7874,34.3535)
High-middle SDI^b^	1,156.32 (872.04, 1,503.31)	492.53 (383.93,633.69)	18.05 (12.64,26.29)	1,666.89 (1,276.64, 2,134.71)	0.3812 (0.2875,0.4956)	0.1624 (0.1266,0.2089)	0.0059 (0.0042,0.0087)	0.5495 (0.4208,0.7037)	5.9006 (5.0191,7.1276)	2.2677 (1.9406,2.7065)	0.1216 (0.1006,0.1483)	8.2899 (7.0768,9.9617)
Middle SDI^b^	1,957.97 (1,503.05,2,482.29)	1,535 (1,196.82,1,957.87)	90.65 (66.06,118.27)	3,583.62 (2,833.22,4,539.24)	0.2613 (0.2006,0.3313)	0.2049 (0.1597,0.2613)	0.0121 (0.0088,0.0158)	0.4783 (0.3782,0.6059)	4.0873 (3.4371,4.8849)	3.3297 (2.83,4.0169)	0.2761 (0.2312,0.3307)	7.693 (6.4974,9.2999)
Low-middle SDI^b^	3,128.6 (2,320.27,4,129.21)	3,064.07 (2,315.95,3,940.74)	225.8 (164.69,302.15)	6,418.46 (4,965.38,8,255.73)	0.4093 (0.3035,0.5402)	0.4009 (0.303,0.5155)	0.0295 (0.0215,0.0395)	0.8397 (0.6496,1.0800)	6.2746 (5.2058,7.5831)	7.3168 (6.2262,8.8061)	0.9809 (0.8301,1.159)	14.5723 (12.3659,17.4166)
Low SDI^b^	1,597.09 (1,212.00, 2,071.77)	1,860.13 (1,429.68, 2,418.71)	152.59 (111.9,200.86)	3,609.82 (2,749.77, 4,653.88)	0.2734 (0.2075,0.3,546)	0.3184 (0.2447,0.414)	0.0261 (0.0192,0.0344)	0.6179 (0.4707,0.7966)	4.4129 (3.6532,5.3955)	5.5897 (4.6711,6.6862)	0.7919 (0.6587,0.964)	10.7945 (9.1163,13.0603)

IBD, inflammatory bowel disease; SDI, sociodemographic index.

^a^ UI is the uncertainty interval, which reflects the certainty of an estimate based on data availability, study size, and consistency across data sources.

^b^ Low SDI, <0.46; low-middle SDI, 0.46–0.64; middle SDI, 0.65–0.74; high-middle SDI, 0.75–0.85; high SDI, >0.85.

**Figure 4:**
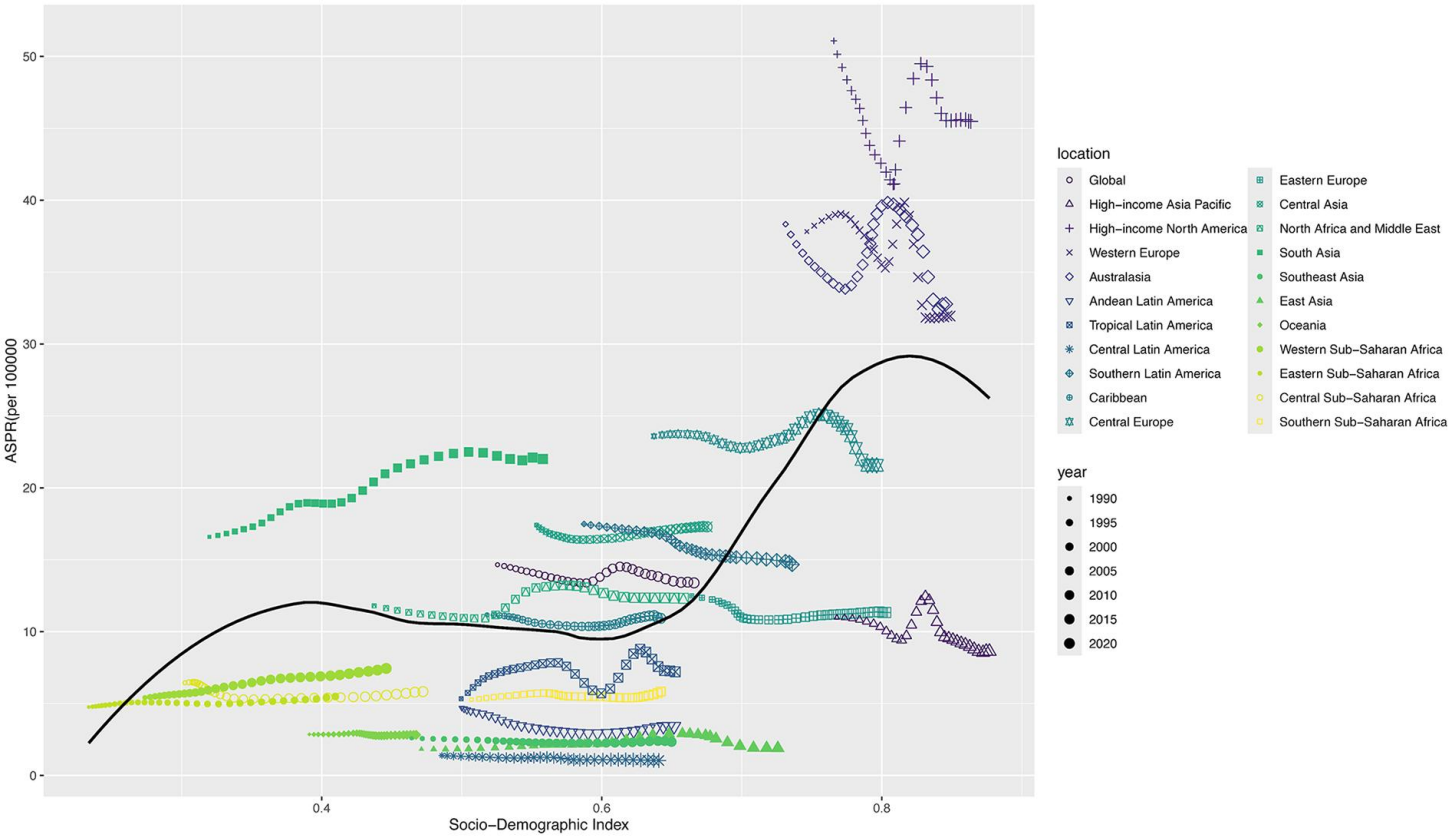
ASPR for children and adolescents of IBD-associated anemia in 22 different SDI regions from 1990 to 2021. For each region, the points from left to right depict ASPR from each year from 1990 to 2021.

Figure 4 shows the ASPR (per 100,000 population) of 22 different SDI regions from 1990 to 2021. In general, as the SDI increased, the ASPR also increased, especially in the high-income areas, including Western Europe, North America, and Australasia. ASPR showed an upward trend from 2005 to 2010 and began to decline from 2011 to 2021 (Figure 4). The Spearman's rank correlation between 22 different SDI regions and ASPR was 0.34 (*P* < 0.001). The distribution of ASPR due to IBD-associated anemia exhibited a similar pattern in 204 countries compared with 22 different SDI regions in 2021 (Supplementary Figure S2), and Spearman's rank correlation was 0.57 (*P* < 0.001).

### 3.3 Trends in prevalence by Joinpoint regression analysis

**Figure 5:**
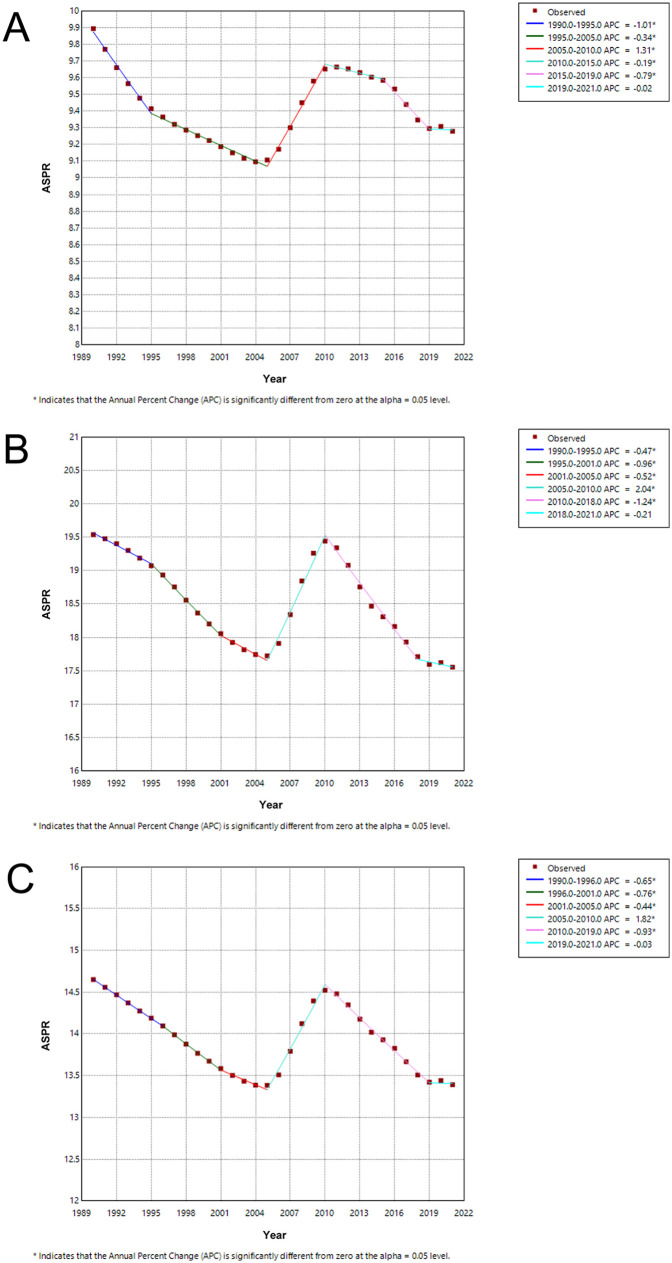
Joinpoint regression analysis of ASPR for children and adolescents with IBD-associated anemia globally from 1990 to 2019. **(A)** Joinpoint regression analysis of ASPR for males. **(B)** Joinpoint regression analysis of ASPR for females. **(C)** Joinpoint regression analysis of ASPR for both males and females.

We conducted the Joinpoint regression analysis of the sex-specific ASPR for children and adolescents of IBD-associated anemia globally from 1990 to 2021 (Figure 5). The prevalence decreased among females from 1990 to 1995 [APC = −0.47 (95% CI: −0.62, −0.33)], and the trend was more evident between 1995–2001 [APC = −0.96 (95% CI: −1.11, −0.81)] and 2001–2005 [APC = −0.52 (95% CI: −0.86, −0.19)]. However, the prevalence increased significantly among females from 2005 to 2010 [APC = 2.04 (95% CI: 1.83, 2.26)]. From 2005 to 2010, the trend of males decreased by 5 years earlier than that of females. The prevalence rates exhibited a significant downward trend from 2010 to 2021 (APC = −1.24 from 2010 to 2018 and APC = −0.21 from 2018 to 2021). The Joinpoint regression analysis trend of both sexes mirrored that of the female trend (Figure 5 and Supplementary Table S1). For males, in addition to the increasing trend from 2005 to 2010 [APC = 1.31 (95% CI: 1.24, 1.38)], all showed a trend of decline in the rest of the years.

The trend of ASPR significantly decreased from 1990 to 2021 [AAPC = −0.29 (95% CI: −0.34, −0.24)] in both sexes. Interestingly, females [AAPC = −0.35 (95% CI: −0.42, −0.28)] had lower AAPCs for ASPR than males [AAPC = −0.20 (95% CI: −0.22, −0.17)], as shown in Supplementary Table S2.

### 3.4 Age, period, and cohort effects on IBD-associated anemia prevalence

Age, period, and cohort effects on children and adolescents of IBD-associated anemia prevalence rate globally during 1990–2021 using the age, period, and cohort model, and the relevant results are shown in Supplementary Figure S3. The prevalence rate showed an increasing trend in the longitudinal and cross-sectional age curves (Supplementary Figures S3A,B).

The period from 2004 to 2009 is taken as 1, and the relative prevalence rates are shown for the other time periods. The period effects revealed an increased risk of IBD-associated anemia prevalence from 1990 to 2010. However, from 2010 to 2021, the period effect had an inverse impact on the risk of IBD-associated anemia (Supplementary Figure S3C). Age-specific trends were summarized by birth cohort using relative risks, with the 1997 birth cohort as the reference group. Cohort relative risks decreased from 1.0739 (95% CI: 1.0387, 1.1102) in 1977 to 0.9938 (95% CI: 0.9649, 1.0235) in 1987, followed by an increase to 1.1447 (95% CI: 0.8325, 1.5739) from 1997 to 2017 (Supplementary Figure S3D). The values and 95% CI values for each point in Supplementary Figure S3 are presented in Supplementary Table S3.

### 3.5 Drivers of IBD-associated anemia: population growth, aging, and epidemiologic changes

To assess the impact of aging, population growth, and epidemiological changes over the past three decades on the IBD-associated anemia in children and adolescents, we performed a decomposition analysis of prevalence rates (Supplementary Figure S4).

The prevalence of IBD-associated anemia in female children and adolescents was significantly higher than that in males. In females, from 1990 to 2021, population growth, epidemiological changes, and age changes accounted for 115.31%, −26.78%, and 11.47% of the incremental burden of IBD-associated anemia, respectively, indicating that population growth had the most outstanding positive contribution to the increase in prevalence. In males, from 1990 to 2021, population growth, epidemiological changes, and age changes accounted for 87.53%, 9.61%, and 2.86% of the incremental burden of IBD-associated anemia, respectively, and all three had positive contributions (more details in Supplementary Table S4). Overall, aging and population growth have been the primary drivers of changes in IBD-associated anemia prevalence globally throughout the study period.

### 3.6 Trends in prevalence of IBD-associated anemia over the next 20 years

**Figure 6:**
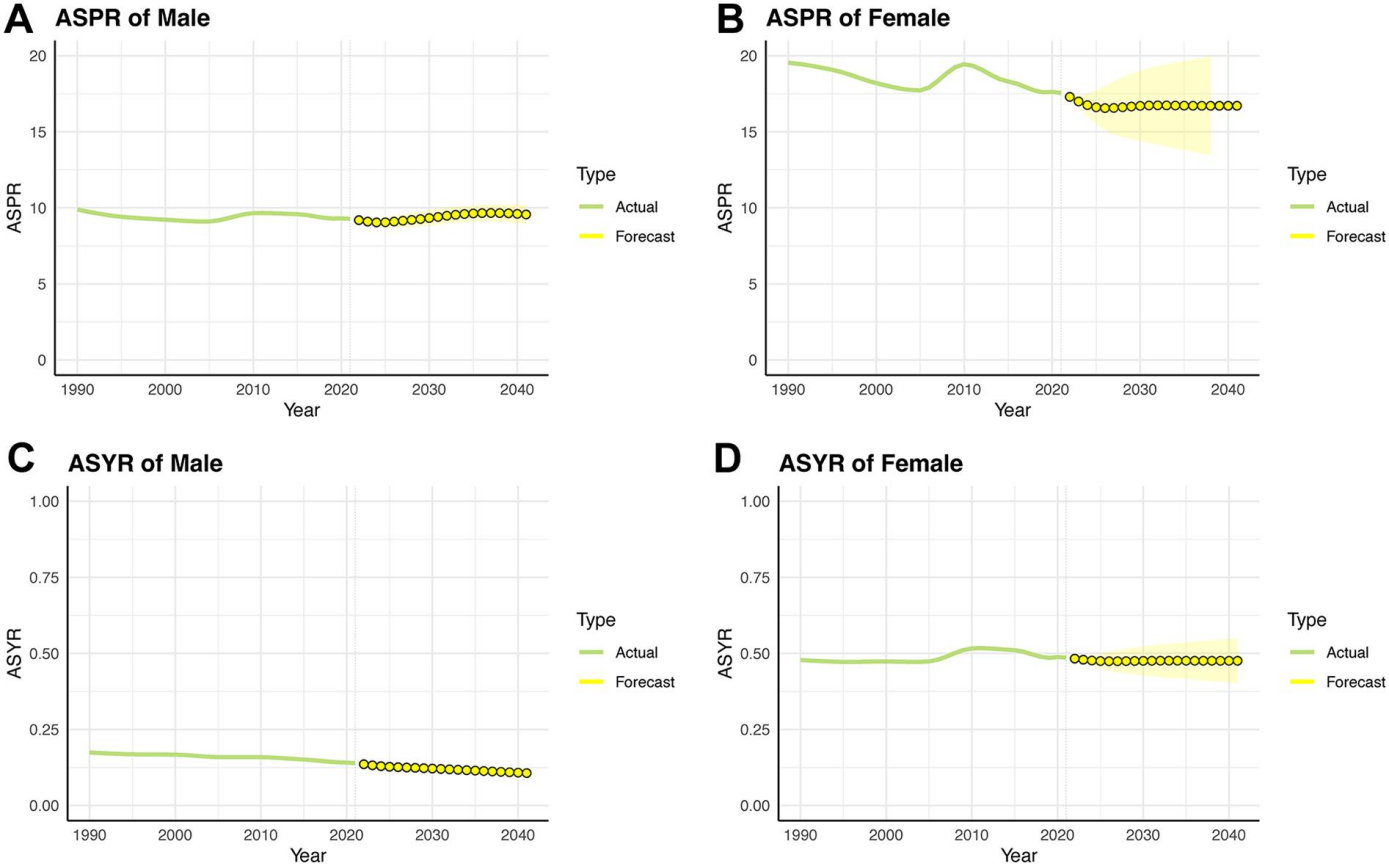
Trends in ASPR and ASYR for children and adolescents of IBD-associated anemia prevalence rate over the next 20 years by the ARIMA model. **(A)** ASPR for males. **(B)** ASPR for females. **(C)** ASYR for males. **(D)** ASYR for females.

The ASPR of IBD-associated anemia in male children and adolescents experienced a dynamic change of decreasing from 1990 to 2004 (ASPR: 9.89–9.10), increasing from 2005 to 2011 (ASPR: 9.10–9.66), and decreasing from 2012 to 2021 (ASPR: 9.65–9.28). We predict that the decreasing trend will change to an increasing trend from 2027. After a sustained decline from 2010 (ASPR: 19.44) to 2027 (ASPR: 16.57), the ASPR among females is projected to remain stable until 2041 (ASPR: 16.71). It is expected that YLDs for IBD-associated anemia will continue to slowly decline in both male and female children and adolescents over the next 20 years (Figure 6 and Supplementary Table S5).

## 4 Discussion

Several studies have suggested a higher prevalence of anemia, mainly iron deficiency anemia, in children with IBD than in adults. Impaired growth and development due to anemia can lead to delayed puberty, which has profound effects throughout a child's life. As far as we know, this is the first study to describe the prevalence and YLDs of IBD-associated anemia in children and adolescents aged <19 years from 1990 to 2021 at global, regional, and country levels. Understanding these trends was critical for policymakers to adequately address this public health concern, early intervention, and treatment, as well as reduce disparities.

Our study found that anemia associated with IBD was more prevalent in those older than 10 years of age, increased rapidly between 15 and 19 years old, and the prevalence was significantly higher in females than in males among children and adolescents. For different gender and age groups, YLDs rate similar trends. IBD-associated anemia was mainly mild and moderate anemia, and ASPR was highest in moderate anemia. The prevalence of IBD-associated anemia associated with national SDI generally represents a positive relationship in children and adolescents. Countries with the low-middle and low SDI quintile exhibited a lower rate compared with high-SDI countries. Joinpoint regression analysis showed that the ASPR of IBD-associated anemia in both males and females increased from 2005 to 2010 and then decreased after reaching a peak in 2010. Aging and population growth have been the primary drivers of changes in IBD-associated anemia prevalence globally throughout the study period. Finally, we predicted the trends in the prevalence of IBD-associated anemia over the next 20 years.

Some studies have reported the prevalence of anemia in children during the diagnosis of IBD and the follow-up of treatment. According to the WHO definition of anemia, different studies have reported a prevalence of 44%–74% at diagnosis and 25%–58% at follow-up 1 year later (7, 8, 27–30). A Netherlands study found that among children with newly diagnosed IBD, the prevalence of anemia was 78%, of which 58% were iron deficiency anemia (IDA) alone (30). They found persistent anemia in one-third of patients at follow-up 1 year after diagnosis. A retrospective cohort study from the UK reported that the prevalence of anemia decreased from 75% among children with newly diagnosed IBD to 42% at 1 year and 32% at 2 years (7). A cross-sectional observational study from the UK reported that in children (3–17 years), the prevalence of anemia was higher than in adolescents (16–26 years) and adults (18–89 years), with anemia defined according to WHO criteria occurring in 70%, 42%, and 40% of children, adolescents, and adults with IBD, respectively (8). Similarly, six hospitals from Sweden pooled a prevalence of anemia of 55% at diagnosis and 28% at 1-year follow-up, which was obviously different from that in adults (*P* < 0.05) (27). In GBD 2021, ASPR per 100,000 population of IBD-associated anemia in children and adolescents was 13.3914% in 2021. The ASPR is 9.2794% for males and 17.5561% for females. Inconsistencies due to differences in data collection methods and time bias among different countries and regions may cause our results to be underestimated.

According to ARIMA projections, ASPR for males is in constant small fluctuations over time, while for females it is stable after 2027. However, the ASPR of IBD-associated anemia was higher in males than in females each year. This difference may be caused by differences between males and females in environmental determinants derived from biological, social, and economic exposures. Alternatively, some studies point to the influence of hormones on the brain–gut–microbiota as the cause of sex differences in IBD prognosis, but the complex pathophysiological mechanism is still not fully understood.

While mortality from chronic diseases such as IBD is decreasing, the incidence in developing countries has a slight increase, which has a dramatic impact on nearly 75% of the world's population (20). Low-middle and low-SDI quintile countries exhibited a lower rate compared with high-SDI countries. This is still consistent with the current epidemiological trend (3–5). The prevalence of IBD-associated anemia in newly industrialized countries such as South Asia, Western Sub-Saharan Africa, Eastern Sub-Saharan Africa, and Central Sub-Saharan Africa has increased rapidly in the past two decades. In terms of our findings, the main drivers of changes in the prevalence of IBD-associated anemia are aging and population growth globally. Notably, the public health impact of IBD is not limited to its burden on the health care system, and the cost of rehabilitation for children and adolescents is also increasing.

IDA, as a chronic disease, is usually ignored by patients (31). Oral iron is the preferred treatment modality for children with mild-to-moderate anemia, and when there are apparent symptoms or life-threatening severe anemia or acute severe hemorrhage, blood transfusion is usually used to correct anemia in patients with IBD (32). It has been shown that, although the number of patients who received blood transfusions was similar to the number who received intravenous iron, there was a significant upward trend in the number of patients who received intravenous iron and a significant downward trend in the number of patients who received transfusions during the study period (31). Even when transfused to correct severe anemia, iron supplementation is required as well as transfusion because the transfused iron cannot be used for further hematopoiesis until the transfused blood cells have been broken down and recovered, a process that can take up to 3 months.

A single-center study found that only 13% of children received oral iron for anemia in IBD, but none received intravenous iron, compared with 30% of adolescents and 41% of adults (8). A retrospective study based on the Pediatric Health Information System database showed that 10% of children with IDA were treated with intravenous iron, and the intravenous iron to correct IDA in pediatric patients with IBD remains low (16). Large published trials in adults with IBD have shown that intravenous iron is a safe, effective, and well-tolerated intervention to correct IDA and maintain iron stores (33, 34). According to the European Crohn and Colitis Organization consensus guidelines, patients with clinically active disease, hemoglobin <10 g/dL (this is 2 g/dL below the lower limit of normal hemoglobin for age in children), or patients with IBD who have a history of intolerance to oral iron should be considered for first-line treatment of iron deficiency (17). Our findings suggest that health policymakers should develop simple, cost-effective, community-based interventions to prevent and treat IBD-associated anemia and reduce the incidence of growth and developmental disabilities in children. Screening for anemia should be performed routinely in all children diagnosed with IBD, every 3 months in active disease, and every 6−12 months in remission or mild disease. Screening for anemia should include at least a complete blood count, C-reactive protein, and ferritin levels. Iron-rich foods and a balanced diet should be recommended to all children with IBD (35).

Our study has some limitations. First, we showed the assessment of IBD in children and adolescents, correlating anemia prevalence and burden of disease according to age, sex, and the degree of anemia, but the incidence of anemia and adjusted life years statistical data are lacking in the database, thus failing to comprehensively evaluate IBD-associated anemia incidence and disease burden. In addition, we were unable to obtain details on screening and diagnostic criteria for anemia in different countries and regions. Furthermore, the GBD database does not have detailed data on whether enrolled patients took iron supplements or any other drug to correct anemia, as well as other treatments that may have affected anemia, which may have biased the results. In addition, because the GBD database is not classified into two subtypes of IBD, UC and CD, we cannot conduct further analysis in this study, which limits the study's pathophysiological specificity and conclusions on the burden of disease. Finally, although GBD 2021 has used all available data to the best of its ability, the data are not yet available for many countries in low- and middle-income countries. In addition, differences and inconsistencies in data collection tools and methods across time periods and countries may affect temporal trends and geographic differences, which may cause our results to be underestimated, so caution is needed when interpreting the results.

## 5 Conclusion

Our study showed that from 1990 to 2021, the prevalence of IBD-associated anemia in females was significantly higher than that in males in children and adolescents worldwide, and the ASPR of IBD-associated anemia in both males and females increased from 2005 to 2010 and then decreased after 2010. The prevalence of IBD-associated anemia in children and adolescents is positively correlated with the national SDI. The prevalence of IBD-associated anemia is predicted to remain stable in children and adolescents over the next 20 years. In the future, IBD-associated anemia will still be an essential public health problem that will incur a lot of health and economic costs.

## Data Availability

The datasets presented in this study can be found in online repositories. The names of the repository/repositories and accession number(s) can be found in the article/Supplementary Material.
