# Supplementary figures and images for: Epidemiology of inflammatory bowel disease-associated anemia in children and adolescents from 1990 to 2021: a global, regional analysis from the Global Burden of Disease Study
Source: Front Pediatr. 2025 Oct 30;13:1651612. doi: 10.3389/fped.2025.1651612 (PMC12611943; doi:10.3389/fped.2025.1651612)

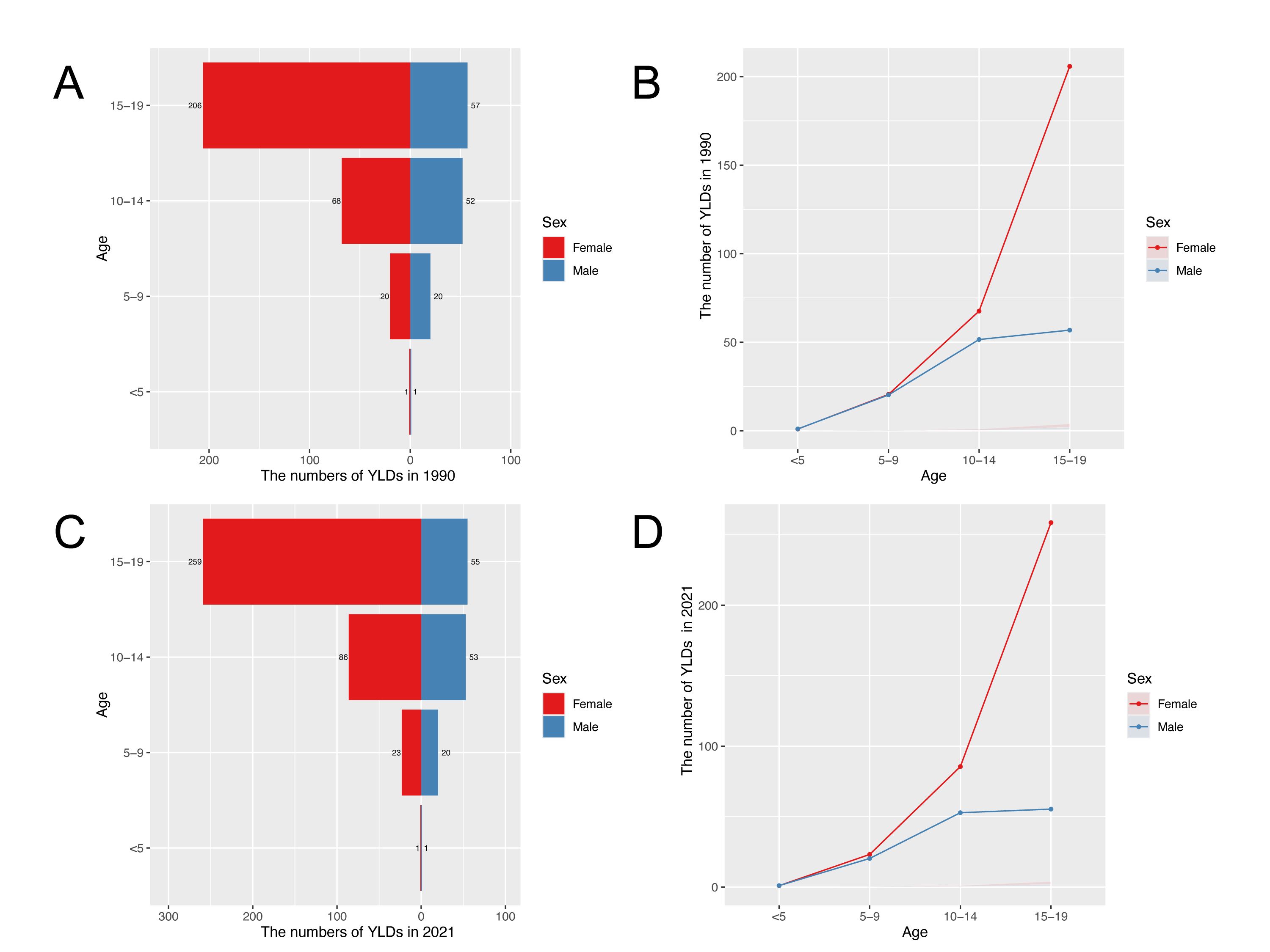

Supplement: Supplementary file 6 [file Image1.tif]

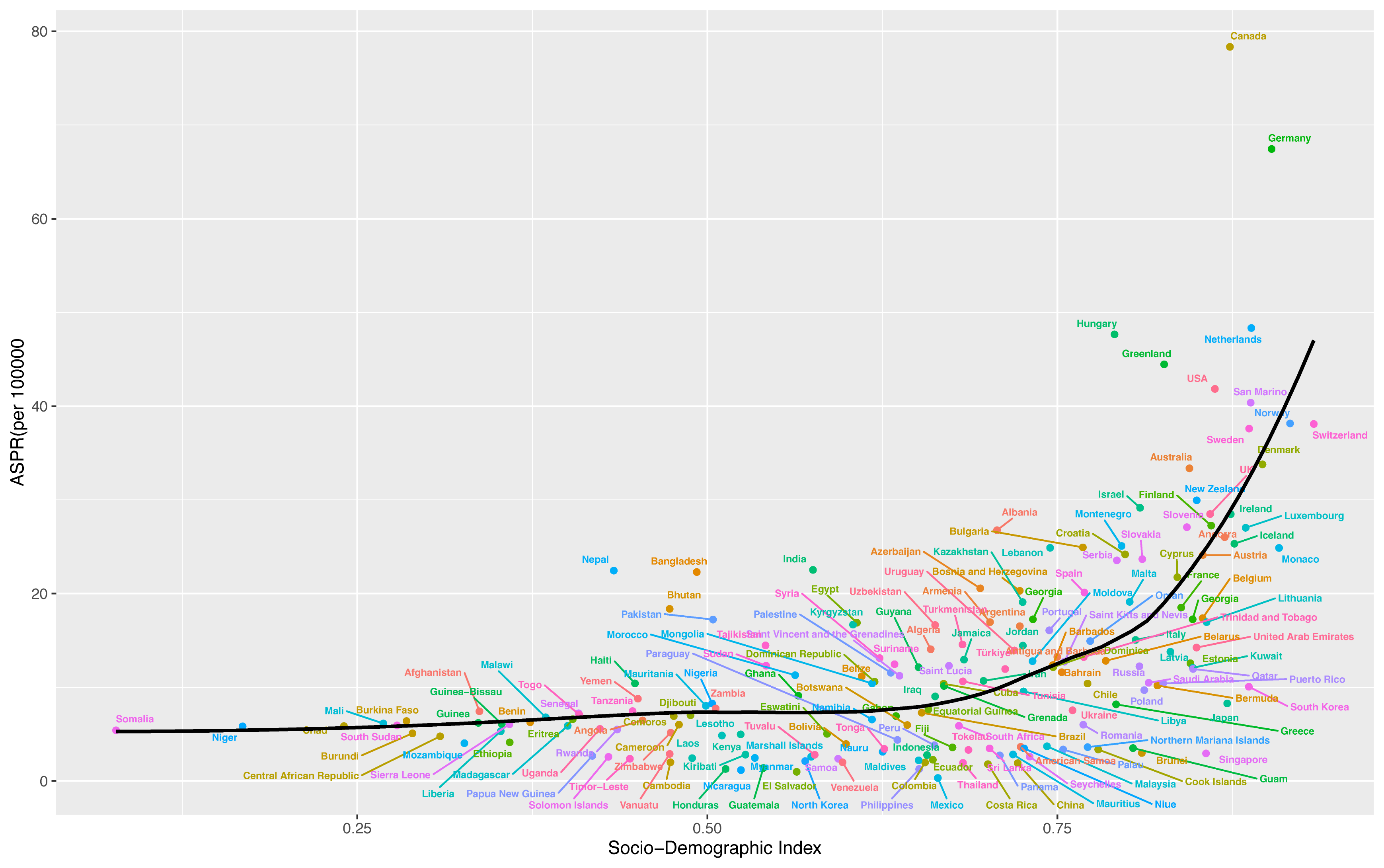

Supplement: Supplementary file 7 [file Image2.tif]

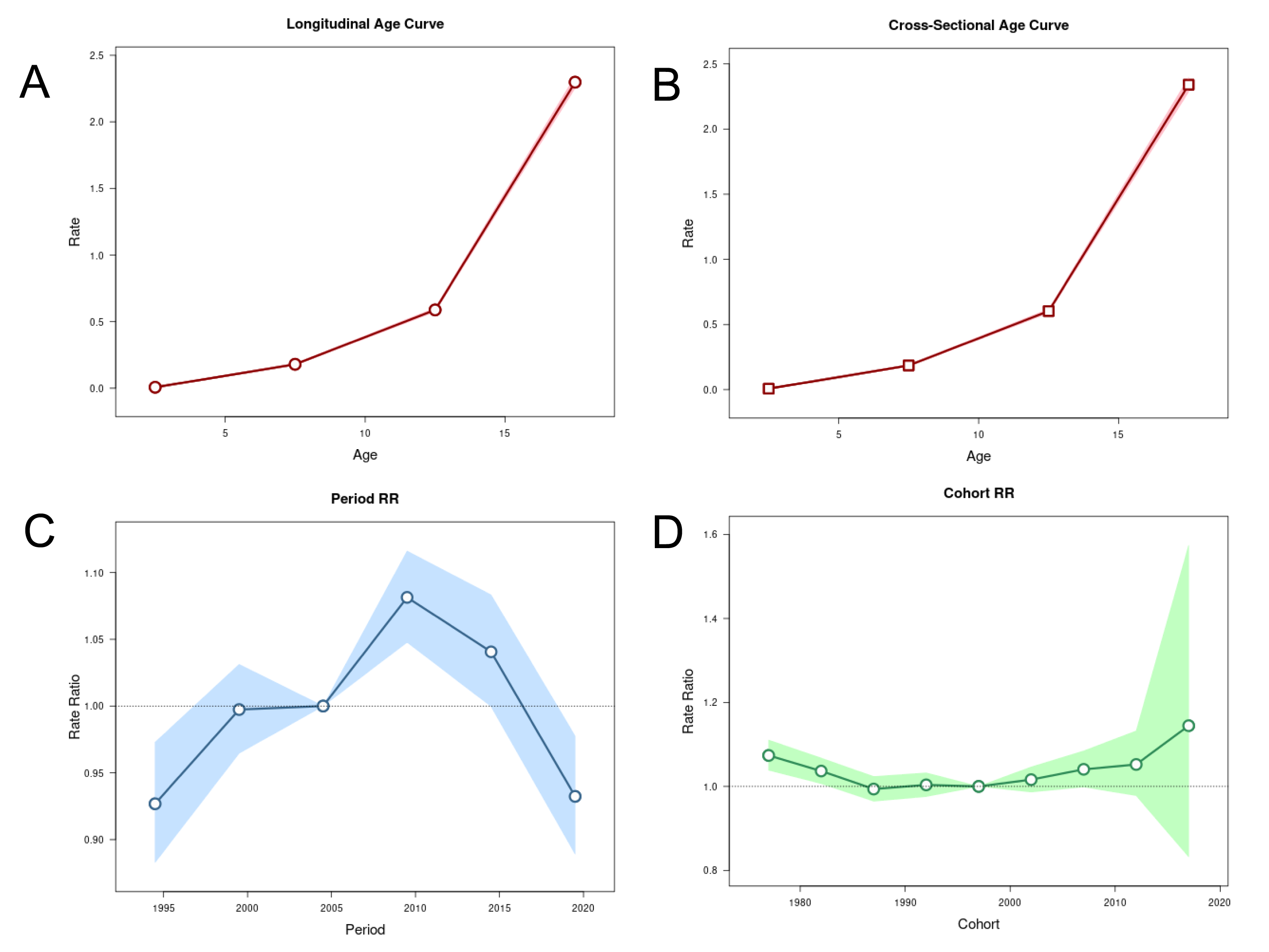

Supplement: Supplementary file 8 [file Image3.tif]

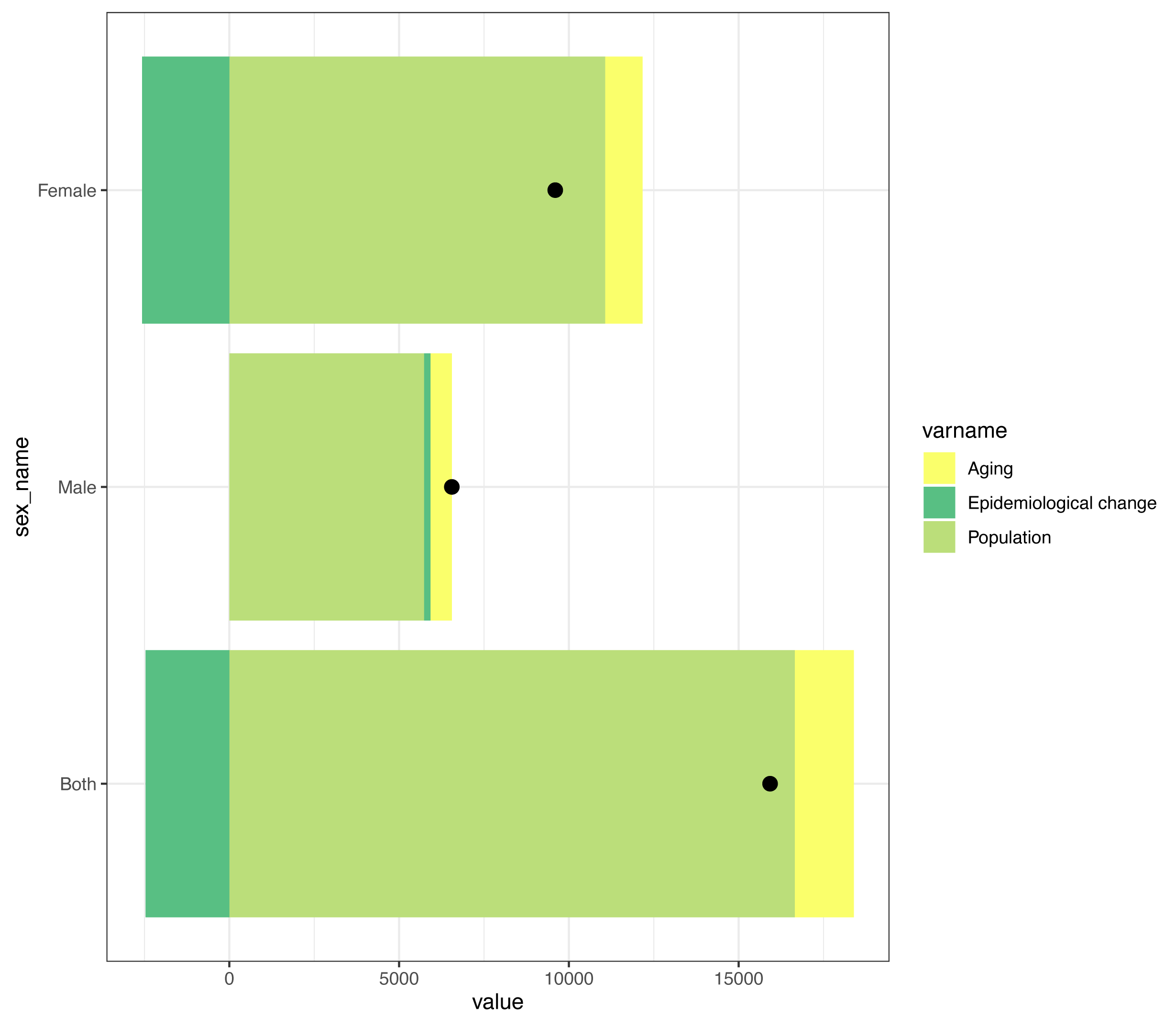

Supplement: Supplementary file 9 [file Image4.tif]
